# Acupuncture Attenuated Inflammation and Inhibited Th17 and Treg Activity in Experimental Asthma

**DOI:** 10.1155/2015/340126

**Published:** 2015-11-03

**Authors:** Ying Wei, Ming Dong, Hongying Zhang, Yubao Lv, Jiaqi Liu, Kai Wei, Qingli Luo, Jing Sun, Feng Liu, Fei Xu, Jingcheng Dong

**Affiliations:** ^1^Department of Integrative Medicine, Huashan Hospital, Fudan University, 12 Middle Urumqi Road, Shanghai 200040, China; ^2^Institute of Integrated Traditional Chinese and Western Medicine, Fudan University, 12 Middle Urumqi Road, Shanghai 200040, China; ^3^Gumei Community Health Center, 668 Longming Road, Shanghai 201102, China

## Abstract

Acupuncture is an effective therapeutic method in asthma treatment in traditional Chinese medicine. Here, we evaluated the effect of acupuncture on airway hyperresponsiveness (AHR) and the associated inflammatory changes as well as Th17 and Treg activity in ovalbumin- (OVA-) induced experimental asthma. Our results revealed that acupuncture treatment significantly inhibited AHR, lung inflammation, and mucus secretion of experimental asthma mice. Furthermore, a decrease in lymphocytes and eosinophils as well as neutrophils was observed in bronchoalveolar lavage fluid (BALF) of mice treated with acupuncture. Acupuncture reduced the OVA specific IgE level as well as the Th17 cytokine levels including IL-17A, IL-17F, and IL-22 in the serum of the experimental asthma mice. Acupuncture treatment group also had reduced CD4+IL-17A+ cell numbers and increased CD4+Foxp3+ cell numbers in BALF. In addition, acupuncture could inhibit IL-17R, ROR*γ*t, p65, and the inhibitor of NF-*κ*B kinase-*α* (IKK*α*) protein expression. Our results indicated that acupuncture was effective in inhibiting AHR and inflammation in OVA-induced experimental asthma, which may be associated with the regulation of Th17 and Treg activity and NF-*κ*B pathway.

## 1. Introduction

Asthma is an inflammatory disease of the airway with airway inflammation, remodeling changes, and airway hyperresponsiveness (AHR) as its key features, affecting approximately 300 million individuals worldwide [1–3]. It is increasingly clear that asthma is also a heterogeneous disease, resulting from genetic, environmental, epigenetic, and other factors [4]. In addition to the classic T-helper (Th) 2 cells dominating in the asthma pathogenesis, Th17 cells which are a recently discovered subtype of T-helper cells have also been involved in the asthma inflammation according to the recent studies. It has been shown that Th17 cells contribute to the asthma disease pathology [5] and control many aspects of the disease through secretion of IL-17 and IL-22, which are frequently found in the airways of mouse model of asthma or in humans with asthma [6–8]. The regulatory T cells (Tregs) are suppressive immune cells essential for inducing and maintaining immunological tolerance to foreign and self-antigens. Tregs are apparently altered in number and function in allergic asthmatic patients, and treatments targeted to ameliorate asthma symptoms result in an increase in Tregs, indicating that these cells play a critical role in the attenuation of inflammation in asthma [9–11]. Furthermore, it has been shown that Tregs have the ability to inhibit Th17 cells function [12, 13].

The inhaled corticosteroids and long-acting bronchodilators represent the milestone of asthma controller therapy and are the most effective antiasthma drugs available. However, there are still concerns about the use of these drugs because of the fear of long-term side effects. In addition, epidemiological and clinical evidences point to the fact that some other asthma phenotypes may not respond well to the present therapies [14, 15], such as Th17 dominated asthma phenotype. Inadequate treatment strategies are most likely due to the complex heterogeneous nature of asthma. One problem we need to face is the limited options for prevention or cure of asthma.

Acupuncture is an effective treatment for asthma in traditional Chinese medicine (TCM). There have been evidences revealing that acupuncture is able to control asthma symptoms and regulate immune responses [16]. Acupuncture is involved in effective regulation of the gene expression of immune response and steroid hormone [17]. Furthermore, it has been identified that electroacupuncture is prominent in promotion of CD4+CD25+Foxp3+ Tregs in an OVA-induced experimental model [18]. However, there is limited evidence of the effect of acupuncture on Th17 activity in asthma. In this study, we sought to examine whether acupuncture was effective in the regulation of inflammation and Th17 and Treg functions in asthma.

## 2. Materials and Methods

### 2.1. Reagents

Ovalbumin (OVA) and methacholine (Mch) were purchased from Sigma-Aldrich. Sterile acupuncture needles (13 mm long, 0.25 mm in diameter) were purchased from Suzhou Shenlong Medical Apparatus Co., Ltd. Mouse Th17 cytokine Bio-Plex kit was purchased from Bio-Rad Laboratories. Mouse IL-17A ELISA kit was purchased from Anogen biopharmaceutical company. Mouse OVA specific IgE ELISA kit was purchased from Shibayagi. Anti-mouse GAPDH and HRP-conjugated IgG were purchased from KangChen Bio-Tech. Anti-mouse IL-17R, ROR*γ*t, Foxp3, p65, and IKK*α* antibodies were purchased from Abcam and Cell Signaling Technology. FITC-labeled anti-mouse CD4, PE-labeled anti-mouse ROR*γ*t, and Alexa Fluor 647-labeled anti-mouse Foxp3 were purchased from BD Pharmingen.

### 2.2. Animals

Specific-pathogen-free BALB/c mice (female, six weeks old, 12 g~15 g) were purchased from Shanghai SLAC Laboratory Animal Co., Ltd., and housed under pathogen-free conditions with air conditioning and a 12 h light/dark cycle with food and water freely available. Forty mice were randomly divided into four groups (10 mice/group), including the normal control (NC), OVA-induced asthma model (A), acupuncture treatment (AA), and sham acupuncture (ASA) groups. The protocol of the study was approved by the Committee on the Ethics of Animal Experiments of Fudan University.

### 2.3. Experimental Asthma Establishment and Treatment

**Figure 1:**
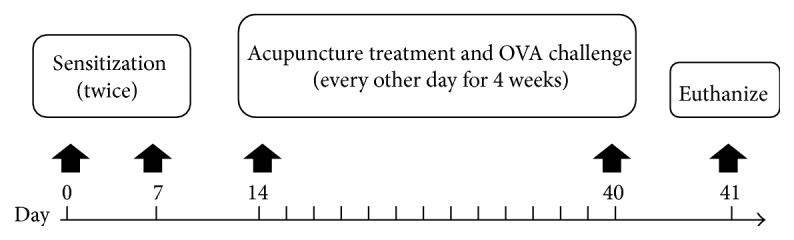
Protocol for experimental asthma and acupuncture treatment.

OVA and alum were used to induce mouse asthma model. As presented in Figure 1, mice were sensitized with 50 *μ*g OVA absorbed with 1 mg alum per mouse in 0.2 mL normal saline on day 0 and day 7. From day 14, mice were placed in a Plexiglas chamber and challenged with 3% OVA solution (w/v) for 30 min every other day for 4 weeks using an ultrasonic nebulizer (402AI, Yuyue Medical Equipment Company, Jiangsu, China). Mice in the NC group were sensitized and challenged with normal saline with the same method.

To perform the acupuncture treatment, the acupoints of GV14 (Dazhui), bilateral BL12 (Fengmen), and BL13 (Feishu) were selected based on the theory of TCM [16, 17]. For mice in ASA group, the distal irrelevant acupoints of bilateral GB30 (Huantiao) were selected for sham acupuncture. From day 14, both acupuncture and sham acupuncture procedures were performed 1 h before each challenge with a fixation device in an awakened state. Sterile needles were inserted into the acupoints with a depth of approximately 3 mm and withdrawn after the needle retaining time of 30 min. During the needle retaining time, no manual manipulations were performed. Mice were sacrificed within 24 h after the last OVA challenge and treatment.

### 2.4. AHR Measurement

Within 24 hours after the final OVA challenge, mice were subjected to measurements of airway responsiveness to inhaled Mch with an invasive method under anaesthesia using Buxco pulmonary system. Increasing doses of Mch (3.125, 6.25, and 12.5 mg/mL) were used to assess airway resistance (*R*_*L*_) of mice in each group. Results were expressed as the percentage change in baseline following Mch challenge.

### 2.5. Leucocyte Count and Classification in Bronchoalveolar Lavage Fluid

Bronchoalveolar lavage fluid (BALF) was obtained and centrifuged at 800 g for 10 min. The cell pellets were resuspended for inflammatory cell count and flow cytometry assay separately. The lymphocyte (Lym), eosinophil (Eos), and neutrophil (Neu) in BALF were counted by Hemavet 950 instrument (Drew Scientific Group).

### 2.6. Histopathological Assessment

The lung tissue was removed after BALF collection, fixed in 4% paraformaldehyde, embedded in paraffin, cut into 5 mm sections, and stained with Hematoxylin and Eosin (H&E). The inflammatory changes were observed in the perspective of 10 times with an optical microscope.

### 2.7. OVA Specific IgE and Th17 Cytokines Determination in Serum

The blood was collected and thereafter centrifuged for serum collection. The levels of OVA specific IgE and Th17 cytokines in serum were determined using Bio-Plex and ELISA assays according to the manufacturer's instructions.

### 2.8. Flow Cytometric Analysis

The cell pellets in BALF were resuspended with 100 *μ*L PBS for further flow cytometry assay. Cells were stained with FITC-labeled anti-mouse CD4 antibody for 30 min at 4°C. After incubation with the fixation/permeabilization solution for 1 h, the cells were stained with PE-labeled anti-mouse ROR*γ*t and Alexa Fluor 647-labeled anti-mouse Foxp3 antibodies for 40 min, followed by detection with a FACSCalibur instrument (BD Bioscience).

### 2.9. Western Blot Assay

The total protein of the lung tissue in each group was extracted according to the manufacturer's instructions. SDS-PAGE was performed using 20 *μ*g protein, and then the targeted proteins were transferred to PVDF membranes and blocked. Thereafter, the targeted proteins were blotted using specific antibodies including anti-mouse IL-17R antibody (1 : 1000 diluted), ROR*γ*t antibody (1 : 1000 diluted), Foxp3 antibody (1 : 1000 diluted), p65 antibody (1 : 1000 diluted), IKK*α* antibody (1 : 1000 diluted), GAPDH antibody (1 : 5000 diluted), and HRP-conjugated secondary antibodies (1 : 10000 diluted). The immunoreactivity was detected by chemiluminescence and quantified by Bio-Rad Image Lab software.

### 2.10. Data Analysis

Data was presented as means ± standard deviation. Statistical significance of the differences were performed by one-way analysis of variance (ANOVA), followed by LST or Games Howell as appropriate. A *p* value < 0.05 was considered statistically significant.

## 3. Results

### 3.1. Acupuncture Suppressed AHR in Experimental Asthma

**Figure 2:**
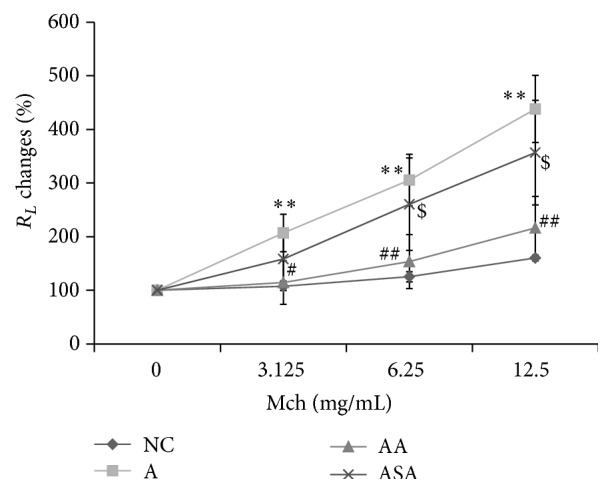
Acupuncture suppressed AHR in experimental asthma. *R*_*L*_ changes to Mch were assessed in each group. Data was expressed as means ± SD values. ^*∗∗*^ *p* < 0.01 versus NC group, ^#^ *p* < 0.05 versus A group, ^##^ *p* < 0.01 versus A group, and ^$^ *p* < 0.05 versus AA group.

In our experiment system, with the increasing dose of Mch administration, mice in OVA-induced experimental asthma group demonstrated increased *R*_*L*_ as compared to mice in NC group (Figure 2, *p* < 0.01). Acupuncture treatment decreased the *R*_*L*_ significantly with the Mch dose increasing compared to the asthma mice (Figure 2, *p* < 0.05 or *p* < 0.01). However, changes in *R*_*L*_ of mice in sham acupuncture group were not obvious compared to the asthma mice (Figure 2, *p* > 0.05). What is more, effect of acupuncture on inhibition of *R*_*L*_ was prominent than that of sham acupuncture with Mch dose increasing (Figure 2, *p* < 0.05).

### 3.2. Acupuncture Reduced Inflammation in Experimental Asthma

**Figure 3:**
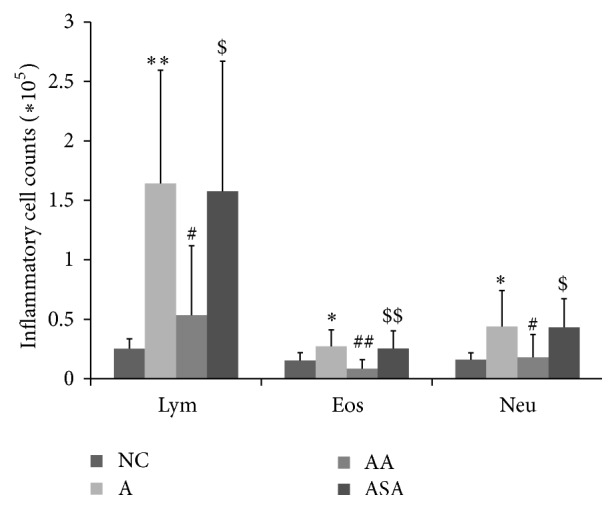
Acupuncture treatment inhibited inflammatory cell levels in BALF of experimental asthma. Data was expressed as means ± SD values. ^*∗*^ *p* < 0.05 versus NC group, ^*∗∗*^ *p* < 0.01 versus NC group, ^#^ *p* < 0.05 versus A group, ^##^ *p* < 0.01 versus A group, ^$^ *p* < 0.05 versus AA group, and ^$$^ *p* < 0.01 versus AA group.

**Figure 4:**
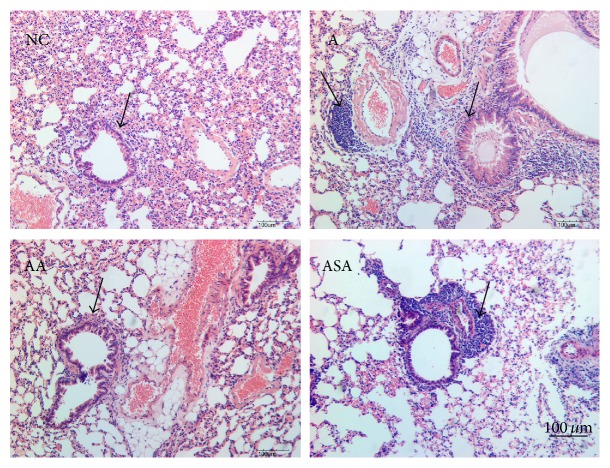
Acupuncture reduced inflammation in experimental asthma. The inflammatory changes of the lung tissue in each group were measured by H&E staining. The arrows indicated the changes in inflammation and mucus secretion in each group. Bar = 100 *μ*m.

The lung inflammation was observed by inflammatory cell counts in BALF as well as H&E staining of the lung slices separately. OVA challenge increased lymphocytes (Figure 3, *p* < 0.01), eosinophils (Figure 3, *p* < 0.05), and neutrophils (Figure 3, *p* < 0.05) recruitment into the lung tissue compared to NC group. Acupuncture significantly reduced these cells' recruitment (Figure 3, *p* < 0.05 or *p* < 0.01). However, these cell numbers did not reduce in sham acupuncture group compared to the asthma mice (Figure 3, *p* > 0.05). H&E staining results revealed that there were a large number of inflammatory cells infiltrating around the airway and plenty of mucus secreted inside the airway of the asthma mice (Figure 4). Acupuncture treatment attenuated the inflammatory cells infiltration and mucus secretion around and inside the airway (Figure 4). Mice in sham acupuncture group did not manifest improvement in lung inflammation (Figure 4).

### 3.3. Acupuncture Regulated Th17 Cytokine Levels in Serum of Experimental Asthma

**Figure 5:**
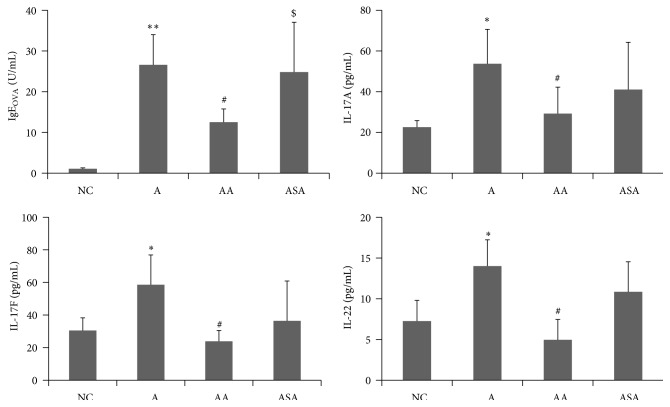
Acupuncture regulated OVA specific IgE level and Th17 cytokine levels in serum of experimental asthma. Data was expressed as means ± SD values. ^*∗*^ *p* < 0.05 versus NC group, ^*∗∗*^ *p* < 0.01 versus NC group, ^#^ *p* < 0.05 versus A group, and ^$^ *p* < 0.05 versus AA group.

Th17 cells were reported to be increased in asthma [5, 19]. We investigated the effect of acupuncture on OVA specific IgE level as well as the Th17 cytokines in the serum of each group. Our results demonstrated that the OVA specific IgE level in serum increased markedly in experimental asthma group compared to mice in NC group (Figure 5, *p* < 0.01). There was a significant reduction in OVA specific IgE level of mice treated with acupuncture (Figure 5, *p* < 0.05). Sham acupuncture did not have any effect on OVA specific IgE level compared to the experimental asthma group (Figure 5, *p* > 0.05). Th17 cytokines which included IL-17A, IL-17F, and IL-22 elevated obviously in asthma group compared to NC group (Figure 5, *p* < 0.05), and acupuncture exerted inhibitory effect on these cytokine levels (Figure 5, *p* < 0.05). However, sham acupuncture did not have such suppressive effect on these cytokines (Figure 5, *p* > 0.05).

### 3.4. Acupuncture Regulated Th17 and Treg Numbers in BALF of Experimental Asthma

**Figure 6:**
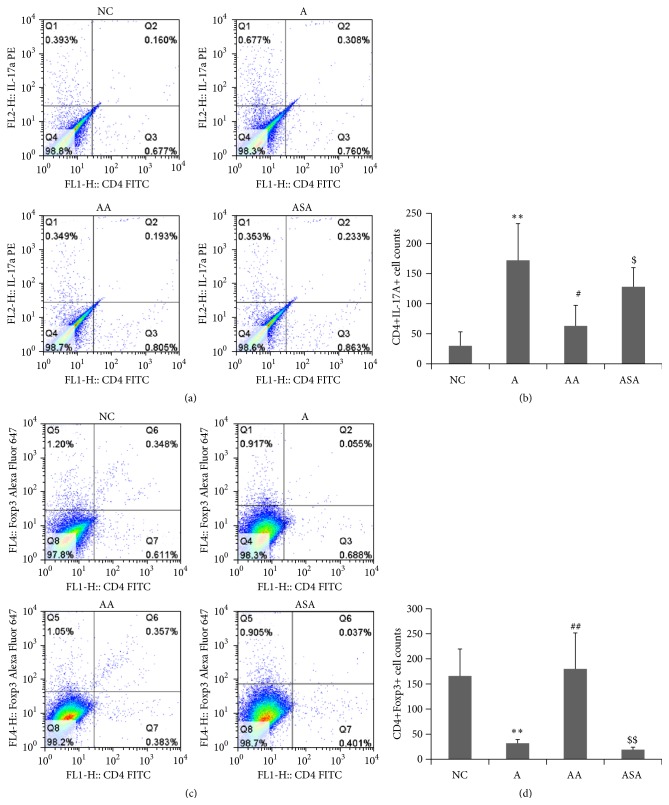
Acupuncture regulated Th17 and Treg numbers in BALF of experimental asthma model. (a, b) Changes in proportion and counts of CD4+IL-17A+ cells. (c, d) Changes in proportion and counts of CD4+Foxp3+ cells. Data was expressed as means ± SD values. ^*∗∗*^ *p* < 0.01 versus NC group, ^#^ *p* < 0.05 versus A group, ^##^ *p* < 0.01 versus A group, ^$^ *p* < 0.05 versus AA group, and ^$$^ *p* < 0.01 versus AA group.

The experimental asthma mice had higher CD4+IL-17A+ cell numbers (Figures 6(a) and 6(b)) and decreased CD4+Foxp3+ cell numbers (Figures 6(c) and 6(d)) in BALF as compared to NC mice (*p* < 0.01). After acupuncture treatment, the AA mice had an obvious reduction in CD4+IL-17A+ cell numbers (Figures 6(a) and 6(b)) and a prominent increase in CD4+Foxp3+ cell numbers (Figures 6(c) and 6(d)) in BALF (*p* < 0.05 or *p* < 0.01). There were not obvious changes in these cell populations in sham acupuncture group compared to the asthma model group (Figure 6, *p* > 0.05). What is more, significant differences in the effect of acupuncture on these cell populations were observed compared to the sham acupuncture (Figure 6, *p* < 0.05 or *p* < 0.01).

### 3.5. Acupuncture Regulated Th17 Associated Factors Expression in Lung Tissue of Experimental Asthma

**Figure 7:**
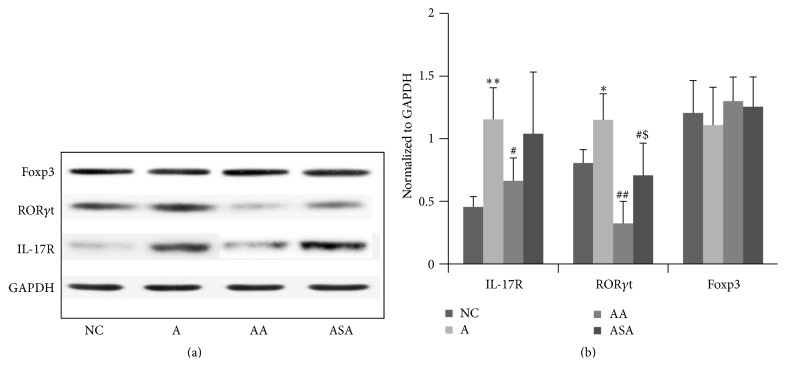
Acupuncture regulated Th17 associated factors expression in the lung tissue of experimental asthma. Data was expressed as means ± SD values. ^*∗*^ *p* < 0.05 versus NC group, ^*∗∗*^ *p* < 0.01 versus NC group, ^#^ *p* < 0.05 versus A group, ^##^ *p* < 0.01 versus A group, and ^$^ *p* < 0.05 versus AA group.

We investigated the protein expression of IL-17R and transcription factors of Th17 and Treg in each group. The IL-17R and Th17 transcription factor ROR*γ*t expression increased obviously in the asthma model group (Figure 7, *p* < 0.01 or *p* < 0.05). Acupuncture treatment resulted in significant reduction in IL-17R and ROR*γ*t protein expression (Figure 7, *p* < 0.05 or *p* < 0.01). Furthermore, sham acupuncture was also effective in lowering ROR*γ*t protein expression, which needs further investigation. However, there were not marked changes in Treg transcription factor Foxp3 expression in each group (Figure 7, *p* > 0.05).

### 3.6. Acupuncture Suppressed NF-*κ*B Expression in Lung Tissue of Experimental Asthma

**Figure 8:**
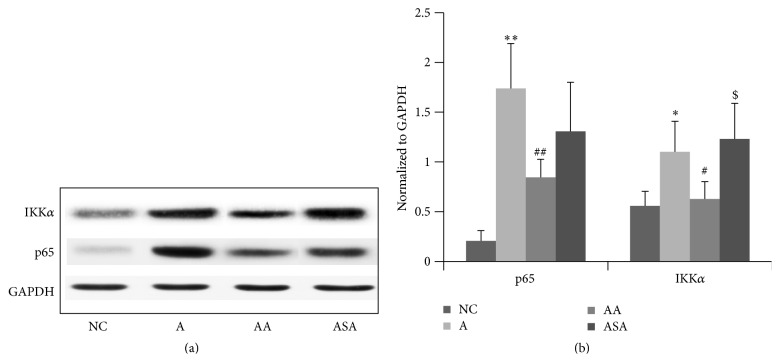
Acupuncture regulated the NF-*κ*B expression in the lung tissue of experimental asthma. Data was expressed as means ± SD values. ^*∗*^ *p* < 0.05 versus NC group, ^*∗∗*^ *p* < 0.01 versus NC group, ^#^ *p* < 0.05 versus A group, ^##^ *p* < 0.01 versus A group, and ^$^ *p* < 0.05 versus AA group.

NF-*κ*B was involved in the differentiation of Th17 cell [20, 21]. We further observed whether acupuncture was effective in regulation of NF-*κ*B activity, and our data revealed that OVA inhalation induced upregulation of the NF-*κ*B p65 and IKK*α* expression (Figure 8, *p* < 0.01 or *p* < 0.05). A decline in the p65 and IKK*α* expression was observed in the acupuncture treated mice (Figure 8, *p* < 0.01 or *p* < 0.05). However, sham acupuncture did not show any obvious effect on NF-*κ*B expression compared to the asthma mice (Figure 8, *p* > 0.05).

## 4. Discussion

Asthma is characterized by inflammatory changes throughout the airways, which is in accordance with our results that acupuncture was effective in the attenuation of AHR and inflammation. Several lines of evidence revealed that an abundance of eosinophils was seen in asthma [22, 23], while other studies identified a more prominent neutrophils infiltration [24, 25]. Our results revealed that OVA challenge caused significant infiltration of both eosinophils and neutrophils in the lungs, which were reversed by acupuncture treatment. Meanwhile, the lymphocytes in BALF were also inhibited by acupuncture, suggesting the anti-inflammatory effect of acupuncture treatment. On the other hand, the large amount of mucus secretion in the airways was also attenuated by acupuncture, indicating the possible inhibitory effect of acupuncture on goblet cell function.

Recent studies had shown that Th17 cells were associated with increased Th17 cytokines in moderate and severe asthma phenotypes [19]. Here, we investigated the effect of acupuncture on these Th17 cytokines, and our results demonstrated that OVA inhalation led to increased levels of IL-17A, IL-17F, and IL-22, which agreed with the published data that IL-17A, IL-17F, and IL-22 were increased in the BALF and bronchial biopsies of patients with moderate and severe asthma [6, 26]. Acupuncture treatment caused significant reduction in these cytokine levels, which may partly explain the attenuation of inflammation by acupuncture. Mounting evidence supported a role for Th17 cytokines in recruiting neutrophils to the airway by increasing secretion of neutrophilic chemokines [27, 28]. In addition, Th17 cytokines could also induce mucus cell metaplasia, both of which were important for induction of inflammation in asthma. Here, we speculated that the increased number of neutrophils and mucus hypersecretion may result from elevated Th17 cytokines, and acupuncture could reverse these inflammatory responses according to our data. We further investigated the effect of acupuncture on the Th17 cells as well as the suppressive immune cell type Tregs in BALF of experimental asthma. Both proportion and cell counts of CD4+IL-17A+ cells increased and CD4+Foxp3+ cells decreased in asthma model. Acupuncture inhibited counts of CD4+IL-17A+ cells and promoted CD4+Foxp3+ cells, indicating the regulatory effect of acupuncture on immune responses.

IL-17R which responds to IL-17A or IL-17F increased in asthma. IL-17R knockout mice have decreased OVA-induced allergic airway inflammation compared to WT mice [29], indicating the inflammatory role of IL-17R in asthma pathogenesis. ROR*γ*t has been shown to be necessary and sufficient for murine Th17 development [30]. Our data demonstrated a marked reduction in IL-17R and ROR*γ*t expression, suggesting a prominent suppressive effect of acupuncture on Th17 responses. IKK*α* was a key transcriptional regulator of Th17 differentiation [31], which was involved in the maintenance of the activation state of the IL-17a locus. There has been evidence revealing that NF-*κ*B family member p65 is required for the production of IL-17 of T cells [20] and therefore required for Th17 differentiation. To test the involvement of NF-*κ*B pathway in the effect of acupuncture, we determined the protein expression in the lung tissue of mice in each group. Here, we revealed that acupuncture resulted in a significant decline in IKK*α* and p65 expression, which is illustrating in part a possible role of acupuncture in the suppression of Th17 function by the inhibition of NF-*κ*B activity.

It was worth noting that sham acupuncture was also effective in reducing ROR*γ*t protein expression, which played critical role in the differentiation of Th17 cells. However, the CD4+IL-17A+ cell numbers in BALF and the inflammatory Th17 cytokines were not inhibited by sham acupuncture, indicating that sham acupuncture did not have the ability to regulate the Th17 cell functions regardless of its inhibition of ROR*γ*t protein expression.

In summary, our results indicated that acupuncture protected from AHR and inflammation in OVA-induced experimental asthma, which may be associated with the regulation of Th17 and Treg activity and NF-*κ*B pathway.
